# A Review on Factors Influencing the Fermentation Process of *Teff* (*Eragrostis teff*) and Other Cereal-Based Ethiopian Injera

**DOI:** 10.1155/2022/4419955

**Published:** 2022-03-24

**Authors:** Yizengaw Mengesha, Alemu Tebeje, Belay Tilahun

**Affiliations:** ^1^University of Gondar, Institute of Biotechnology, Department of Agricultural Biotechnology, Ethiopia; ^2^University of Gondar, Institute of Biotechnology, Departments of Industrial Biotechnology, Ethiopia

## Abstract

Fermented foods and beverages are the product of the enzymaticcally transformed food components which are acived by different microorganisms. Fermented foods have grown in popularity in recent years because of their alleged health benefits. Biogenic amines, bioactive peptides, antinutrient reduction, and polyphenol conversion to physiologically active chemicals are all possible health benefits of fermentation process products. In Ethiopian-fermented foods, which are mostly processed using spontaneous fermentation process. Injera is one of the fermented food products consumed in all corners of the country which sourdough fermentation could be achieved using different LAB and yeast strains. Moreover, the kind and concentration of the substrate and the type of microbial flora, as well as temperature, air supply, and pH, all influence the fermentation process of injera. This review article gives an overview of factors influencing the fermentation process of teff ('Eragrostis tef.') and other cereal-based Ethiopian injera.

## 1. Introduction

Fermentation is an old food preparation technique that can be found all around the world [1]. Fermentation procedures are also shown to remove undesired components, improve the nutritious content, flavor, and taste of the food, and make the product safe from pathogenic microbes. They are also the most cost-effective technique of manufacturing and storing food [2, 3]. Modern beverages and fermented food items are a significant part of the food of both industrialized and evolving countries [4]. Microorganisms play a key role in physically and nutritionally changing plant and animal constituents to produce these modern foodstuffs. Kandasamy et al. [5] describe how many types of yeast prevail in African raw plant and animal ingredients. Specific yeast species (*Pichia fermentans*, *Pichia occidentalis*, *Candida humilis*, *Saccharomyces cerevisiae*, and *Kazachstania bulderi*) tend to have a greater product-specific predominance. Microorganisms convert raw ingredients into appealing foods with improved shelf life and protection, both biochemically (nutrients) and organoleptically (taste/texture/odor and visual appearance) [6]. Furthermore, food microbiota improves nutritional value (vitamins, fatty acids, essential amino acids, and others), digestibility, and pharmacological values [7].

Ethiopians are consmud a varied range of beverages and fermented foods derived from a diverse range of basic materials, the majority of which naturally ferment. Injera, kocho, tella, awaze, borde, and tej are just a few of the traditional fermented foods prepared and consumed in Ethiopian communities [8]]. In Ethiopia, injera is a thin, soft fermented baked food made from cereal flour that is fermented for 24 to 96 hours, depending on the optimum temperature. Sorghum, teff, corn, wheat, and barley are among the cereals used in its preparation [9]. The majority of Ethiopians made injera from their own harvested teff ('Eragrostis tef.') far more frequently than from any other source [10]. Sour tastes, smooth, moist, elastic, spongy feel, multiple “eyes” (bubbles), and a long shelf life are the indicators of quality injera [11].

Many bacterial and yeast species are involved in the fermentation of injera teff dough, demonstrating the delicate nature of injera dough fermentation. According to the different studies, a varied microbial community is used to establish the starter culture for injera fermentation and other traditional food processes [12]. The inclusion of beginning culture in the fermentation process improves process efficiency, reduces organoleptic variability and uniformity, increases product shelf life, and improves microbiological quality [13]. To execute controlled fermentation, pure or mixed starting cultures, as well as appropriate technology, can be used. Yeasts and lactic acid bacteria are common starting cultures with proven health advantages [14].

However, fermented food products have little consistency with regard to the final product due to a lot of factors. Temperature, pH, aeration, substrate concentration, and nutrient availability all influence the fermentation process and metabolic processes. The objective of this review is to summarize the major factors that influence the fermentation process of teff and other cereal-based Ethiopian injera.

## 2. Fermented Foods

“Foods or drinks generated through regulated microbial growth and the enzymatic activity to convert dietary components” are the definitions for fermented diets. Fermented foods and drinks are tasty and nutrient-dense foods created from raw or heated raw materials [15]. They are noted for their flavor, aroma, and texture, as well as their superior cooking and processing abilities. Microorganisms contribute to the creation of distinctive qualities like taste, scent, visual appearance, texture, shelf life, and safety through their metabolic activities [16].

Enzymes indigenous to the raw materials may play a role in enhancing these characteristics. Through trial and error, traditional skills have been developed for controlling technical parameters during fermentation processes [17]. Experience has also shown that backloping, or the inoculation of raw materials with a residue from a previous batch, accelerates the initial phase of fermentation and results in the promotion of desirable changes during the fermentation process [18]. Fermented foods can be found in almost all cuisines. Fermented foods have grown in popularity in recent years in the west, owing to a variety of factors, including potential health benefits and an increased interest in gastrointestinal health. Fermented foods may benefit your health and help you fight disease in a variety of ways. To begin with, probiotic microorganisms such as LABs play a significant role in fermentation starting processes [19].

Most fermented items contain at least 10^6^ microbial cells per gram, with concentrations varying based on numerous factors such as the product's geography, age, and time of analysis/consumption [20]. The surrounding food matrix appears to play a critical role in probiotic strain survival by buffering and guarding against intestinal conditions (e.g., low pH and bile acids). According to numerous studies, microbes from fermented foods can penetrate the gastrointestinal tract. However, this varies by product, and their presence in the gut appears to be transitory [21]. These microorganisms may still be able to provide a physiological advantage in the gut by competing with harmful bacteria and producing immune-regulatory and neurogenic fermentation by-products [22].

Second, fermentation-derived metabolites may be helpful to one's health. Polyamines and bioactive peptides produced by lactic acid bacteria, for example, may have an impact on cardiovascular, immunological, and metabolic health (applicable to both non-dairy-fermented and dairy foods) [23].

Finally, fermentation has the potential to transform some chemicals into physiologically active metabolites. Lactic acid bacteria, for example, can convert phenolic substances (like flavonoids) into physiologically active metabolites [24]. In the end, food components contained in fermented foods and drinks, for example, prebiotics and vitamins, may be beneficial to one's health [25]. Lastly, fermentation can treat individuals with properly functioning bowel disorders like digestive disorders, which mitigate ingredients better by reducing toxins and antinutrients. Fermentation lowers phytic stomach acid, and sourdough processing lowers fermentable carbohydrate concentrations (e.g., fermentable oligosaccharides, disaccharides, simple sugars, and polylactic acid), which may assist people with gastrointestinal illnesses [26].

Meat and fish, dairy, vegetables, soybeans, other legumes, grains, and fruits are just a few examples of items that have been fermented in the past. The microbes, nutritional elements, and climatic circumstances all have a role in the fermentation process, resulting in hundreds of different types of fermented foods [27]. Food fermentation has long been used as a technique of preservation since the production of antimicrobial metabolites (e.g., organic acids, ethanol, and bacteriocins) minimizes the likelihood of pathogenic microorganism contamination [28]. Some foods, such as olives, are inedible without fermentation that eliminates bitter phenolic chemicals; hence, fermentation is employed to improve the organoleptic qualities (e.g., taste and texture) [29].

Fermentation of foods can be accomplished in two ways. For starters, foods can be fermented spontaneously, also known as the “wild fermentation process” [30] or “spontaneous yeast,” in which the bacteria are organically present in fresh food or manufacturing environments, such as sauerkraut, kimchi, and some fermented soy products. To produce ethanol and carbon dioxide, yeasts consume carbohydrates such as fructose and glucose in the starter. Many bacteria are toxic to ethanol (i.e., alcohol), but lactic acid bacteria are not bothered by it. In fact, certain lactic acid bacteria can survive higher quantities of ethanol than yeast. Although bacteria prefer maltose, yeast and bacteria do not have to compete for the same meal. Other bacteria that cannot withstand acidic surroundings cannot develop because of the acid created by the bacterium. In turn, yeast that thrives in acidic settings breaks down starches into simpler carbohydrates that bacteria and yeast can consume more easily. Second, foodstuffs can be fermented by using a fermentation process, which is referred to as “culture-dependent ferments,” such as kefir, kombucha, and natto. Natural (e.g., backslapping) or selected pharmaceutical beginnings could be employed to induce fermentation and standardize the organoleptic qualities of the product. One method of doing a culture-dependent ferment is “backslopping,” which involves introducing a little piece of a previously fermented batch to uncooked food, such as sourdough bread [31].

### 2.1. Traditional Fermented Foods

The majority of Ethiopians make traditional fermented foods from a variety of ingredients like teff, sorghum, enset, and milk. Injera, kocho, siljo, and ergo are traditional fermented meals made from these primary components. Kocho is composed up of fermented food created from the pseudostem, pulverized corm, and decorticated pulp, and it is high in starch [32].

Fermented foods are created by utilizing a range of common materials and bacteria in a variety of ways all around the world. Fermentation products include alcoholic beverages, lactic acid, acetic acid, and alkaline fermentation [33]. The most prevalent microorganisms engaged in the alcoholic fermentation process, which produces ethanol, are yeast strains (e.g., wines and beers). Lactic acid bacteria dominate lactic acid fermentation (e.g., fermented milk and grains), whereas Acetobacter species dominate acetic acid fermentation (e.g., fermented milk and cereals). In the fermentation of fish and seeds, both of which are often used as condiments, alkali fermentation is widespread [34].

### 2.2. Ethiopian Traditional Fermented Foods

Ethiopia is one of the countries that consume numerous nonfermented and fermented foods and beverages. Kocho, injera, awaze, tella, tej, and borde are among them. Nonalcoholic fermented foods include bukre, shamita, cheka, korefe, and merissa; and nonfermented foods include kita, guenfo, atmit, and kinche [32]. Lactic acid fermentation, fungal fermentation, and alkaline fermentation are the three types of traditional food fermentation. Kocho is a lactic acid fermented food in southern Ethiopia [35]. Fermented food products provide diet food to consumers, practically utilized in developing countries. In addition, fermented foods are used as nutritious and balanced food, as well as anticancer, antiaging, antiobesity, and anticonstipation [36].

Fermented foods are not only high in nutrients, but they can also help you stay healthy and avoid disease [29]. Lactic acid bacteria and yeasts make up the majority of microorganisms found in fermented foods. At home, you may make traditional fermented dishes and beverages in a variety of ways [37]. Fermented milk, fermented cereals, nonalcoholic beverages, fermented fruits and vegetables, and fermented meat are only a few examples. Traditional fermented foods have existed in human diets since the beginning of time and are widely consumed [38].

Injera is indeed a fermented food produced from a variety of cereals, including sorghum, teff, corn, wheat, and barley, or a mixture of these cereals. Many Ethiopians prefer injera made from teff (Eragrostis tef) to injera made from other grains [39]. Injera is a pancake-like meal made from cereal flour that has been treated to traditional fermentation for 24 to 96 hours, carried out at ambient temperature [40].

## 3. Teff-Based Fermentation

Teff ('Eragrostis tef.') is used to make porridge, kitta (unleavened bread), atmit (gruel), injera (a pancake-like bread) [41], and indigenous alcoholic beverages such as tela, arake, and shamita. Injera, a thin, flat, traditional fermented pancake, is Ethiopia's most widely consumed dish by both young children and adults.

### 3.1. Nutritional Composition of Teff

Teff is as nutritious as, if not more nutritious than, wheat, barley, and maize [42]. Teff grains are high in potassium and phosphorus and include 72.1-75.2 percent carbohydrate, 14-15 percent protein, 11-33 mg iron, and 100-150 mg calcium [43]. Different reports show the nutritional composition of teff, with the greatest and lowest fat content of 4 percent and 2 percent [44], fiber content of 4 percent and 2 percent, carbohydrate content of 74 percent and 68 percent, and moisture content of 13 percent and 10 percent, respectively [45].

**Figure 1:**
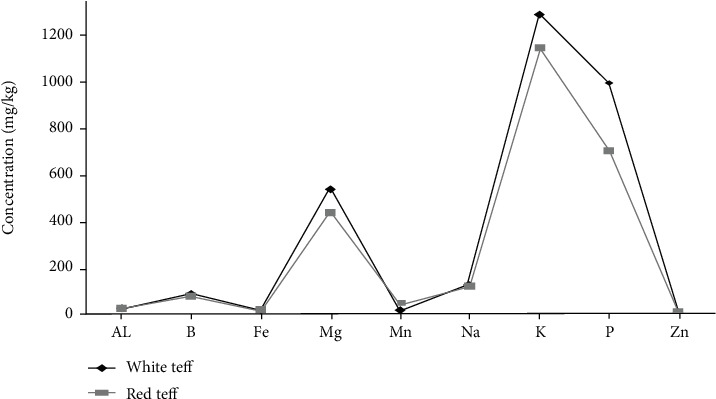
Trace element content of white and red teff grain [50].

Teff has a superior amino acid profile, with lysine levels that are higher than wheat or barley, as well as high calcium, phosphorus, iron, copper, aluminum, barium, and thiamine levels [46]. Redi [47] has challenged the lack of anemia in Ethiopia because of its high iron content (0.05 percent) (Figure 1).

Teff is gluten-free, making it a better choice for people who are allergic to wheat or have gastrointestinal problems [48]. Glutelins and albumins were the most abundant protein storage components in teff [49], with glutelins accounting for 44.55 percent, albumins 36.6 percent, prolamin 11.8 percent, and globulins 6.7 percent, respectively. Though grain proteins possess a small number of prolamins, proteins are meant to be easy to digest [49].

## 4. Injera Fermentation

Injera is a classic Ethiopian and Eritrean ethnic staple dish that is also popular in Somalia [51]. Ethiopian cuisine is based on injera, a fermented pancake-like bread that is prepared from a range of cereals, such as teff, wheat, barley, sorghum, or maize, or a combination of many of these cereals, depending on availability and quantity [52], although it is most usually made from teff. 'Eragrostis tef.' is a kind of teff ('Eragrostis tef.'). When it comes to producing injera, many people believe that teff grain is superior to other cereal grains [53].

Injera goes through two rounds of spontaneous fermentation, which can take anywhere from 24 to 72 hours depending on the ideal temperature [54]. The fermentation container is partially cleaned for backslopping, and milled, sieved teff flour is mixed with water in the fermentation container. Then, ersho is added as an inoculum source and left to ferment for 48 hours. Then, as the first fermentation nears its conclusion, a clear, yellow liquid appears on the surface of the dough [55]. This liquid is thrown away, and a portion of the fermenting dough is cooked by mixing it with water (called “absit” in the area). After the absit has cooled, it is combined with the rest of the dough and allowed to ferment for 35 to 2 hours to raise it [52].

**Figure 2:**
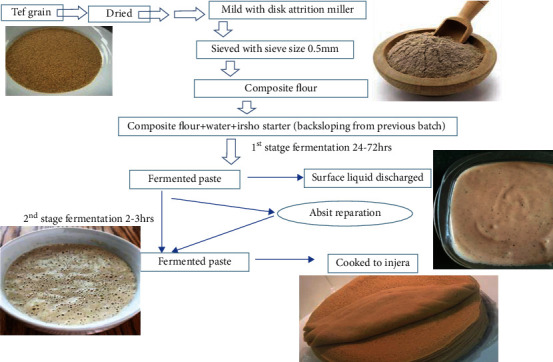
Traditional methods of making injera [56].

The inclusion of absit (portion of the batter mixed with water and boiled) improves the texture, consistency, dough rising, and gas generation processes, but it only takes a few minutes. On the top surface of injera, baked even without absit, there are fewer eyeballs (pits) which shows in (Figure 2) below. The number of larger eyeballs on the upper surface determines the attractiveness of injera [56]. Aflegna is a delicious injera that has been cooked after only 24 hours of fermentation. At this point, the fermenting dough becomes thin enough to be placed on a hot flat pan, locally known as mitad, and steam baked as injera. Overall, the baking duration for one injera is 2–3 minutes [52].

## 5. Injera Sourdough

Sourdough should be considered a unique and stressful ecosystem with variable carbohydrate concentrations, an acidic environment, limited oxygen availability, and higher counts of LAB with 10^8^ colony-forming units per gram (CFU/g) than yeasts (10^7^ CFU/g), resulting in a LAB to yeast ratio of 10 : 1 to 100 : 1 [57]. The sourdough microbiota's metabolic influences include acidification (LAB), taste production (LAB and yeasts), and leavening (yeasts and heterofermentative LAB species). Indeed, the LAB metabolism aims for the rapid depletion of carbohydrate sources and the buildup of organic acids, whereas some yeasts adopt an accumulate-consume metabolic strategy, which is especially noticeable with ethanol and glycerol [58]. As mentioned by [6], different lactic acid bacteria and yeasts are involved in sourdough fermentation.

## 6. Factors Affecting Fermentation Process of Traditional Foods

### 6.1. Aeration

**Figure 3:**
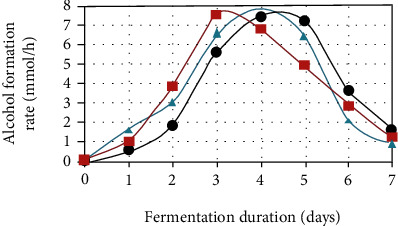
Effect of yeast aeration method on the of ethyl alcohol formation rate [62].

Aeration is an important component that affects the fermentation process of many components. The effects could be the Custer effect, which inhibits growth in anaerobic conditions, or the Pasteur effect, which inhibits fermentation in aerobic conditions. In aerobic conditions, D. bruxellensis is severely suppressed by S. cerevisiae in batch fermentations [59]. The presence of high aeration causes a decrease in the final ethanol yield. In the process, yeast creates acetic acid instead of alcohol, yeast destroys ethanol in the presence of air, or ethanol evaporates when gas exchange is not controlled [60]. Aeration induces acetic acid production and if it affects the total ethanol yield, so limited air is a better fermentation condition to get a good product [60] as shown in (Figure 3). The oxygen supply should be adequate for cell development and maintenance but restricted to avoid excessive cell growth and ethanol absorption. For ethanol production, the amount of oxygen transfer coefficient, oxygen transfer rate, and dissolved oxygen tension should all be restricted. KLa values in the range of 2.3 to 5.9 h1 were regarded as having the optimum balance between productivity and yield. In terms of DOT, *S. stipitis* produced more ethanol when the saturation level was less than 1% [61].

### 6.2. Temperature

Temperature is an important factor for microbial growth; all microbes have a certain optimum range in which they can grow [63]. It is even possible that a higher or lower temperature has an important coeffect with some of the other factors involved. As the temperature rose, the maximum fermentation time decreased, but a much higher temperature inhibited cell growth and then significantly reduced fermentation because the higher temperature changes the transport activity or saturation level of soluble compounds and solvents in the cells, potentially increasing the accumulation of toxins such as ethanol inside cells. The cells, on the other hand, displayed reduced specific growth rates at lower temperatures, which could be due to their low tolerance to ethanol at lower temperatures [64].

**Figure 4:**
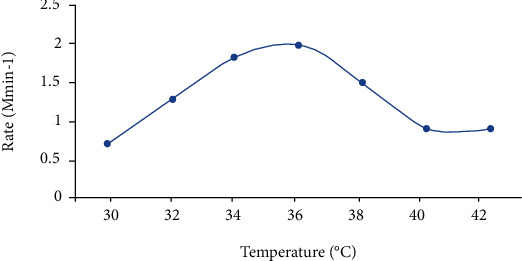
Variation of rate of fermentation with temperature of substrate using 50 (*v*/*v* %) substrate, yeast 1.0 (*w*/*v*%), and pH 5.0 [69].

Fermentation temperature and baking temperature of fermented tef batter may also have an effect on the quality of injera. Cereal mashes with a pH of 5-6.2, which are rich in fermentable carbohydrates, will be preferentially fermented by LAB, at least to a pH below 4, and below this point, acid-tolerant yeasts dominate the fermentation the pH of injera to be 3.4 [65]. Fermentation temperature has been found to impact the pH of spontaneous tef fermentations and quality of injera [66]. Temperature control is critical in sourdough production as changes in fermentation temperature may cause variation in microflora of sourdough and thus variation in sourdough and final bread quality and flavor [67]. The optimum temperature range for yeasts is 20-30°C. Most lactic acid bacteria thrive at temperatures between 18 and 22 degrees Celsius, whereas Lactobacillus species thrive at temperatures above 22 degrees Celsius [68]. Temperatures in Ethiopia's highlands range from 17 to 25 degrees Celsius; therefore, injera produced at these temperatures should still have the desired quality qualities (Figure 4).

### 6.3. pH

**Figure 5:**
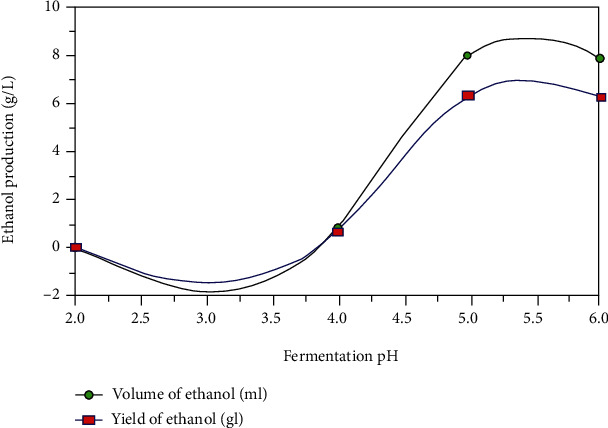
The effect of pH on ethanol production [73].

pH is a component that could affect cell development as well as ethanol yield [70]. The effect on yeast growth is most likely due to changes in toxicity of hydrolysate by-products rather than a pure pH effect on growth since it is not subjected to extreme conditions [71]. Changes in the functional pH of the ethanol production process may utilize the main fermentation pathway. It is critical to maintain a pH range of 4.0 to 5.0, but if it exceeds this range, the production of by-products such as acetic and butyric acid may have eaten part of the substrates, lowering ethanol fermentation efficiency [72] as shown in Figure 5.

### 6.4. Substrate's Type and Concentration

Different fermentation patterns occurred depending on the substrates. According to Assefa [74], the main fermentable sugars in barley-wheat and wheat-red sorghum-injera were maltose and glucose, with a transient accumulation of maltose [74]. The major sugar that transiently accumulated in the teff-white sorghum-injera was glucose, with negligible maltose accumulation. Fermentation trends in BW-and wheat-red sorghum injeras reveal a two-step fermentation process including lactic acid fermentation and alcoholic fermentation, assuming LAB and yeast collaboration.

Substrate concentrations of 20 to 300 kg/m^3^ influence ethanol generation. When the pH was not controlled, greater initial glucose concentrations above 80 kg/m^3^ at 30**°C** necessitated a longer incubation period, while when the pH was controlled [75], a longer incubation duration was required for greater starting glucose concentrations than 80 kg/m^3^ at 30**°C** [76].

Furthermore, when the pH value was not adjusted, larger initial glucose concentrations, such as 300 kg/m^3^, may have reduced ethanol conversion efficiency since the higher substrate and production concentrations may have impeded the ethanol fermentation process.

### 6.5. Fermentation Time

Teff flour and water are mixed in a bohaka, which is a clay, metal, or wood container, to make injera batter in Ethiopia [9]. This mixture is thickened with irsho, a clear yellowish fluid left over from the previous fermentation batch. Depending on the desired injera flavor, the three components are properly blended and the thin, and watery mixture is incubated for 12-72 hours at room temperature. Aflegna injera is characterized by its thickness, sweet flavor, odor, and brownish red bottom and is made from batter that has only been fermented for 12 to 24 hours [77]. Traditional injera is fermented for 48 to 72 hours, and komtata injera is created from an overly fermented batter (usually attributed to the labors of uneducated laborers). Over the course of the fermentation, the teff settles on the bottom of the bohaka, leaving a yellowish or blackish liquid on top [78]. A portion of this irsho is saved for the next batch, and the rest is poured off. About 10% of the fermented paste is mixed with three parts of water and boiled [79]. This boiled batter is called absit, and it is added back to the batter in the bohaka to initiate a second fermentation that lasts 1.5 to 2 hours. Adding absit is critical to developing the desired texture and consistency, as injera made without absit tends to be powdery and have fewer of the “eyes” which are so prized by Ethiopian consumers [9].

### 6.6. Chemical Attributes

In addition to producing acidity, microbiological metabolism has an impact on the chemical composition of the fermenting teff batter. The angular shape of starch granules is damaged during fermentation due to enzymatic breakdown, according to light and electron microscopy study of fermented teff [80]. Fermentation had no effect on bran and embryo fragments, cell walls, or protein bodies, on the other hand. During fermentation, microorganisms created fibrillar threads that bonded flour particles together [81]. Exopolysaccharide, which is produced by lactic acid bacteria, is the most likely source of this fibrillar material.

### 6.7. The Microbiota of Injera Sourdough

A sourdough microbiome is composed of specific yeast species and LAB after it has settled. Yeasts (lower eukaryotes) are facultatively anaerobic, unicellular, acid-tolerant fungi that reproduce sexually without a fruiting body [82]. Yeasts are important in the agricultural and food industries because they either aid biotransformation or cause food and feed to deteriorate. Fermentative, facultative fermentative, or reparative are the three types [83]. The Firmicutes phylum has a varied collection of lactic acid-producing bacteria known as LAB; they are gram-positive, nonsporulating, and exclusively fermentative, and they play a key part in the manufacture of fermented foods. The number of distinct yeast species recovered from a single sourdough is always less than the number of LAB species [84].

Different groups of LAB and yeasts are involved in injera sourdough fermentation. From fermenting teff dough [6], *Enterococcus avium*, *Enterococcus durans*, *Lactobacillus paracasei*, *Enterococcus hirae*, *Lactobacillus brevis*, *Enterococcus faecium*, and *Bacillus subtilis* were also identified. According to various researchers, the microbial ecology of fermenting teff dough was diverse, with *Enterobacteriaceae* and aerobic mesophilic bacteria among the bacteria. Mold, *Enterobacteriaceae*, aerobic mesophilic bacteria, yeasts, fermentative aerogenic, gram-negative bacteria rods, lactic acid bacteria, Bacillus spp., and yeast [32] are among the microbes involved in teff flour fermentation, according to [6].

Strong acid-producing lactic acid bacteria, as demonstrated by Gunduz et al. [85], are members of the genera *Pediococcus*, *Lactobacillus*, *Streptococcus*, *Leuconostoc*, and Bacillus. In addition, Bintsis [86] also showed that different genera of lactic acid bacteria were responsible for the acidic characteristics of the dough, and these included *Pediococcus cerevisiae*, *Lactobacillus brevis*, *Lactobacillus plantarum*, and *Lactobacillus fermentum*.

On the other hand, *Pichia fermentans*, *Rhodotorula mucilaginosa*, *Candida humilis*, *Kazachstania*, *Yarrowia lipolytica*, *Saccharomyces cerevisiae*, and *Pichia occidentalis* were also identified from injera sourdough [87] which indicated that yeast found in dough predominantly consisted of *Candida milleri*, *Rhodotorula mucilaginosa*, *Kazachstania marxianus*, and *Pichia naganishii*. *Candida milleri* was found in over 80% of the samples from every household and R. mucilaginosa, the second most abundant, was encountered only in <40% of the samples. The dominant yeast of fermenting teff dough was species of *Trulopsis*, *Saccharomyces*, *Candida*, and *Pichia*. Neela and Fanta [9] have indicated that *Candida humilis*, *Saccharomyces cerevisiae*, *Cryptococcus tropicalis*, and *Saccharomyces exiguus* were found from the beginning to the end of teff injera batter fermentation.

In a previous study, Parapoul et al. [88] reported that *Candida guilliermondii* was isolated from fermenting teff and that they indicated that it was responsible for starch hydrolysis and an increase in the concentration of reducing sugars in the early phase. Yeasts become the predominant group of organisms in the liquid layer after 50 h of fermentation and after the pH level reaches below 5 [89]. Saccharomyces spp. becomes abundant and is responsible for the rising of the dough during the second stage of fermentation. The yeasts most prevalent in the yellow fluid belonged to the genera *Candida* and *Pichia* [5, 90]. *Saccharomyces* spp. and *Torulopsis* spp. are the dominating flora during the secondary fermentation [91]. All those identified microorganisms may be involved in the fermentation of teff dough during injera preparation. At species level, knowing the microorganisms found in teff dough at species level is important for other aspects like starter culture formulation to solve problems facing teff injera [21, 26].

## 7. Conclusion

Ethiopians consume a varied range of beverages and fermented foods derived from a diverse range of basic materials; the majority is driven from spontaneous fermentation using natural microbiota. Injera is indeed a fermented food produced from a variety of cereals, including sorghum, teff, corn, wheat, and barley, or a mixture of these cereals. Teff is used to make food products including injera and indigenous alcoholic beverages such as tela, arake, gluten-free beer, and shamita. Many Ethiopians prefer injera made from teff (*Eragrostis tef*) than injera made from other grains. pH, substrate concentration, temperature, and aeration are the most important factors for fermentation process of injera. Therefore, this review indicates some overviews of factors affecting injera fermentation and its quality in general.

## Data Availability

The data used to support the findings of this article are available on request from the corresponding author.
